# Characterization of Nucleoside Reverse Transcriptase Inhibitor-Associated Mutations in the RNase H Region of HIV-1 Subtype C Infected Individuals

**DOI:** 10.3390/v9110330

**Published:** 2017-11-08

**Authors:** Sinaye Ngcapu, Kristof Theys, Pieter Libin, Vincent C. Marconi, Henry Sunpath, Thumbi Ndung’u, Michelle L. Gordon

**Affiliations:** 1 HIV Pathogenesis Programme, Doris Duke Medical Research Institute, University of KwaZulu-Natal, Private Bag X7, Congella, Durban 4013, South Africa; Ndungu@ukzn.ac.za (T.N); Tarinm@ukzn.ac.za (M.L.G.); 2 Centre for the AIDS Programme of Research in South Africa, University of KwaZulu-Natal, Private Bag X7, Congella, Durban 4013, South Africa; 3 Rega Institute for Medical Research, Department of Microbiology and Immunology, KU Leuven–University of Leuven, 3000 Leuven, Belgium; kristof.theys@kuleuven.be (K.T.); pieter.libin@vub.ac.be (P.L.); 4 Artificial Intelligence Lab, Department of Computer Science, Vrije Universiteit Brussel, Brussels, 1050 Elsene, Belgium; 5 Division of Infectious Disease, Emory University School of Medicine, Atlanta, GA 30322, USA; vcmarco@emory.edu; 6 Infectious Diseases Unit, Nelson Mandela School of Medicine, University of KwaZulu-Natal, Private Bag X7, Congella, Durban 4013, South Africa; henrysunpath@yebo.co.za; 7 Africa Health Research Institute, University of KwaZulu-Natal, Private Bag X7, Congella, Durban 4013, South Africa; 8 Ragon Institute of MGH, MIT and Harvard University, Cambridge, MA 01239, USA; 9 Max Planck Institute for Infection Biology, Chariteplatz, D-10117 Berlin, Germany

**Keywords:** HIV-1, RNase H, resistance, mutations, NRTIs

## Abstract

The South African national treatment programme includes nucleoside reverse transcriptase inhibitors (NRTIs) in both first and second line highly active antiretroviral therapy regimens. Mutations in the RNase H domain have been associated with resistance to NRTIs but primarily in HIV-1 subtype B studies. Here, we investigated the prevalence and association of RNase H mutations with NRTI resistance in sequences from HIV-1 subtype C infected individuals. RNase H sequences from 112 NRTI treated but virologically failing individuals and 28 antiretroviral therapy (ART)-naive individuals were generated and analysed. In addition, sequences from 359 subtype C ART-naive sequences were downloaded from Los Alamos database to give a total of 387 sequences from ART-naive individuals for the analysis. Fisher’s exact test was used to identify mutations and Bayesian network learning was applied to identify novel NRTI resistance mutation pathways in RNase H domain. The mutations A435L, S468A, T470S, L484I, A508S, Q509L, L517I, Q524E and E529D were more prevalent in sequences from treatment-experienced compared to antiretroviral treatment naive individuals, however, only the E529D mutation remained significant after correction for multiple comparison. Our findings suggest a potential interaction between E529D and NRTI-treatment; however, site-directed mutagenesis is needed to understand the impact of this RNase H mutation.

## 1. Introduction

South Africa is experiencing one of the world’s most devastating HIV-1 epidemics, with an estimated 18.9% of the adult population infected with HIV-1 at the end of 2016 [1]. As over 3 million people had received antiretroviral therapy (ART) by 2016 [1], South Africa has the largest ART programme worldwide where nucleoside reverse transcriptase inhibitors (NRTIs) make up the backbone in recommended first- and second-line regimens [2,3]. While the prevalence of HIV-1 drug resistance is decreasing in developed countries, the up-scale ART in low and middle income African countries will most likely coincide with an increased emergence of HIV-1 drug resistance.

Most HIV-1 drug resistance studies have focused on HIV-1 subtype B, the dominant subtype in developed countries [4,5], but an increased awareness of non-B subtypes in the last decade has shown that HIV-1 subtypes can differ in the mode and tempo of drug resistance pathways [6]. To date, for any subtype, most of the mutations identified to be associated with decreased treatment response have been described for the N-terminal half that corresponds to the polymerase region, up to the first 334 amino acids [7]. In contrast, the C-terminal RT region, encompassing the connection and RNase H domains, has historically been less well characterized in the context of drug resistance [7,8]; however, studies on subtype B-infected individuals suggested that mutations in the RNase H domain can increase NRTI resistance [9,10,11,12,13].

Data indicating whether similar mutations develop under NRTI drug selection pressure in the RNase H domain of HIV-1 non-B subtype viruses is limited [7,8]. Given the increasing accessibility of ART in South Africa, where subtype C prevails, we investigated the emergence of mutations in the RNase H domain of the subtype C virus in the context of during NRTI treatment.

## 2. Materials and Methods

### 2.1. Study Cohort

HIV-1 subtype C sequences spanning the entire RNase H region (RT codons 431–560) were obtained from 112 ART-experienced and 28 ART-naive patients enrolled in the South African Resistance Cohort Study (SARCS), McCord Hospital, and the Paediatric Tropism Study, King Edward VIII Hospital, Durban, South Africa, respectively. The SARCS study was a prospective study assessing the prevalence of drug resistance after virologic failure in patients on combined antiretroviral therapy (cART) between January 2005 and August 2006 [14] and the Paediatric Tropism study was a prospective paediatric study between August 2008 and January 2010 [15]. All ART-experienced patients received two NRTIs either stavudine (d4T) or zidovudine (AZT) plus lamivudine (3TC) and a non-nucleoside reverse transcriptase inhibitor (NNRTI), either efavirenz (EFV) or nevirapine (NVP). Participants who experienced virologic failure were defined according to clinical, immunological, and virological criteria outlined in the South African guidelines for the management of HIV in children and adults [2]. Participants receiving second line ART drug regimen were excluded in this study. This study was approved by the Biomedical Research Ethics Committee of the University of KwaZulu-Natal (Ref: BE 106/09).

### 2.2. Amplification, Sequencing and Phylogenetic Analyses of the RNase H Domain

Viral RNA was isolated from 140 µL of plasma using the QIAamp RNA kit (QIAGEN Services, Inc., Germantown, MD, USA) according to the manufacturer’s instructions. A 469 base pair product was amplified by nested PCR, using primers and PCR conditions as previously described [7]. PCR products were purified using the QiAquick gel purification kit (QIAGEN Inc.) according to the manufacturer’s instructions. Sequences were generated using the inner primers and the BigDye v3.1 cycle sequencing kit (Applied Biosystems, Foster City, CA, USA), and run on an Applied Biosystem (ABI) 3130xl automated sequencer as previously described [7]. Sequences were assembled and edited using the Phred Phrap and Consed software programs (http://www.phrap.org/phredphrapconsed.html) and aligned against HXB2 reference strain [16]. The alignment was manually edited using the Genetic Data Environment v2.2 (http://www.bioafrica.net/GDElinux/GDEmicrobial). HIV-1 subtype was determined by the REGA HIV-1 subtyping tool version 3 [17,18]. A maximum likelihood phylogenetic tree was built with PhyML using the GTR substitution model and 100 bootstraps [19]. In order to investigate a confounding effect of epidemiology on the identification of treatment-associated mutations, a phylogenetic tree was constructed using sequence data from which positions found to be significant in this analysis were removed.

### 2.3. Identification of Treatment-Related Mutations in RNase H of HIV-1 Subtype C

Treatment-related mutations in RNase H were identified by comparing mutation prevalence between viral sequences from ART-experienced patients and sequences from ART-naive patients. Additional subtype C sequences spanning the RNase H domain were obtained from the Los Alamos database, limiting to ART-naive patients from South Africa (*n* = 359) to minimize the epidemiological bias. In addition, a control dataset of subtype B sequences from ART-naive patients (*n* = 1782) spanning the RNase H domain were obtained from the Los Alamos database. Major NRTI resistance mutations were defined according to the Stanford HIV-1 drug resistance database [20]. The Fisher’s exact test was used to detect mutations significantly more prevalent in ART-experienced patients, with a level of significance of 0.05. *p*-Values were adjusted using a False discovery rate step-down procedure to reduce false positive results [21]. Known and novel mutations were identified by comparing with NRTI-associated RNase H mutations previously identified from subtype B drug-resistance studies: K451R, D460N, L469F, T470N, T473A, N474A, Q475A, K476A/N, D488E, L491S, Q500A, Y501A/F, I505A, I506L, Q509L, K512Q, K527N, K530R, H539N, V518I, E529D, Q547K, D549N, A554S and K558E/R [7,8,9,11,12,22,23,24,25,26,27,28,29].

### 2.4. Bayesian Network Construction

Bayesian network learning was applied to identify novel NRTI resistance mutation pathways in RNase H. Mutations identified using the Fisher’s exact test were included together with polymorphisms prevalent over 15% in the ART-naive population. Dependencies between mutations were learned and represented graphically as described previously [30,31,32]. The robustness of the network was assessed with a non-parametric bootstrap using 500 replicates.

## 3. Results

### 3.1. Prevalence of RNase H Mutations in ART-Naive Subtype C Sequences

HIV-1 subtype C viral sequences from 112 NRTI-treated and 28 ART-naive HIV-1 patients in the study cohort were amplified and sequenced. In addition, sequences from 359 subtype C ART-naive sequences were downloaded from the Los Alamos database to give a total of 387 sequences from ART-naive individuals for the analysis. No unusual clustering of the study sequences was observed, confirming that there was no cross-contamination between sequences.

**Figure 1:**
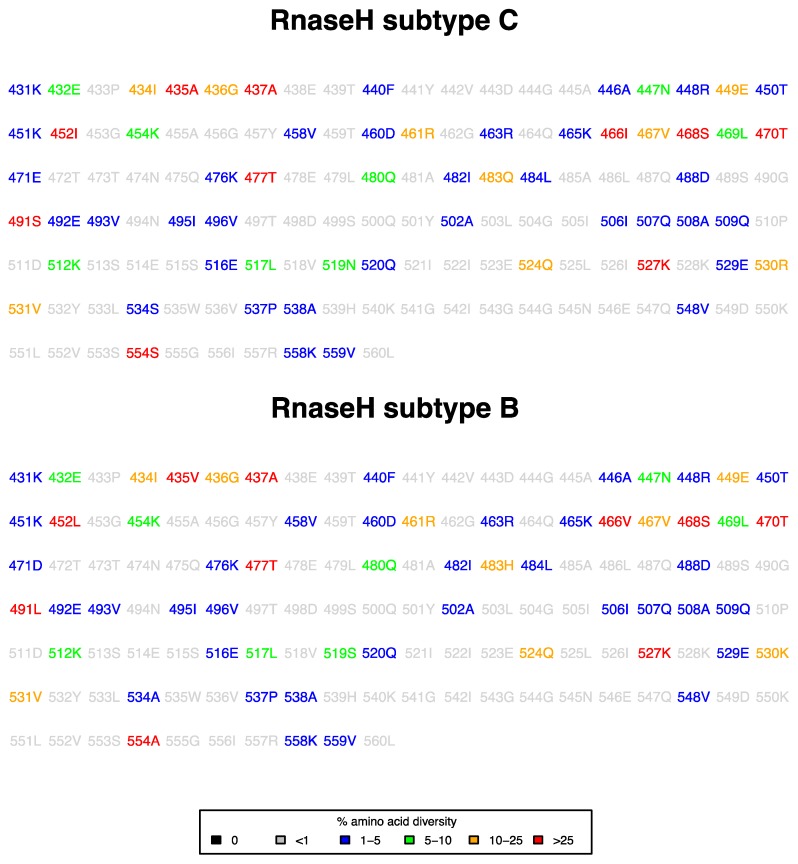
Natural variability in 387 RNAse H subtype C sequences and in 1782 RNAse H subtype B sequences from ART-naive patients. The consensus amino acid at each position is coloured according to frequency.

We assessed the natural variability in the RNase H region of subtype C by determining the amino acid distribution of the 130 positions (from amino acid 431–560) of the ART-naive sequences (Figure 1). The analysed amino acids included the RNase H primer grip (R448, T473, N474, Q475, K476, Q500, Y501 and I505), RNase H active site (D443, E478, D498 and D549) and the RNase H sites overlapping the protease cleavage site (E438, T439, F440, Y441, V442, D443, G444 and A445) [29,30,31,32]. The RNase H primer grip and active site were well conserved, with positions 474 and 475 showing less than 1% variation, and less than 5% variation for positions 448 and 476. Furthermore, the RNase H motif E438-A445 that overlaps the protease cleavage site was well conserved. This agrees with previous studies, suggesting that any amino acid variation at these highly-conserved sites may have functional constrains on the virus in vivo [33,34,35]. The consensus amino acid of subtype C differed at 10 positions in RNase H (435, 452, 466, 471, 483, 491, 519, 530, 534, 554) from the consensus amino acid of subtype B (Figure 1). Interestingly, the consensus amino acid in the wild-type subtype C sequence was associated with drug resistance in subtype B, including S491 (75.2%), K512 (93.4%), R530 (88.9%) and S554 (64.7%). Other subtype B resistance-associated mutations found in the naive subtype C sequences were: T470N (13.6%), Q509L (1.6%), K527N (27.1%) and K558R (3.8%). The full amino acid distribution per position is available as Supplementary Material (Figures S1–S3).

### 3.2. Prevalence of RNase H Mutations in NRTI-Treatment-Experienced Compared to ART-Naive Subtype C Sequences

**Figure 2:**
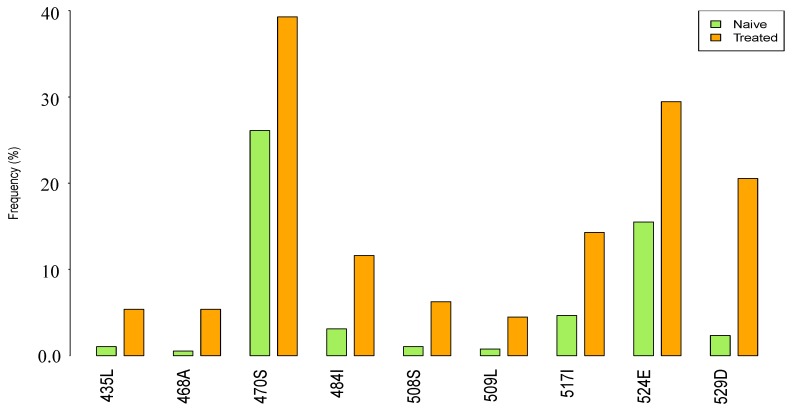
The frequency (%) of treatment-associated mutations in antiretroviral therapy (ART)-naive patients and ART-experienced patients.

We searched for treatment-associated mutations by comparing the prevalence of amino acids at each position between sequences from 387 ART-naive and 112 NRTI-experienced patients. The most frequently observed reverse transcriptase (RT) drug resistance mutations in NRTI-experienced patients were M184I/V (64.2%), D67N (25.9%), K70R (19.6%), K219Q/E (17.9%) and T215Y/F (13.4%). Other known NRTI-elicited mutations were less prevalent (<1%). A total of 9 mutations in RNase H were found positively selected in ART-experienced patients compared to ART-naive patients, these included respectively A435L (5.4% vs. 1.0%), S468A (5.4% vs. 0.5%), T470S (39.3% vs. 26.1%), L484I (11.6% vs. 3.1%), A508S (6.3% vs. 1.0%), Q509L (4.5% vs. 0.7%), L517I (14.3% vs. 4.6%), Q524E (29% vs. 15.5%) and E529D (20.5% vs. 2.3%) (Figure 2). Only E529D remained significant after adjusting for multiple comparisons (*p* < 0.001). In contrast, mutations E432D, A446S and P537Q occurred at a lower frequency in the treatment-experienced versus naive sequences.

### 3.3. Dependencies between RNase H domain Mutations

**Figure 3:**
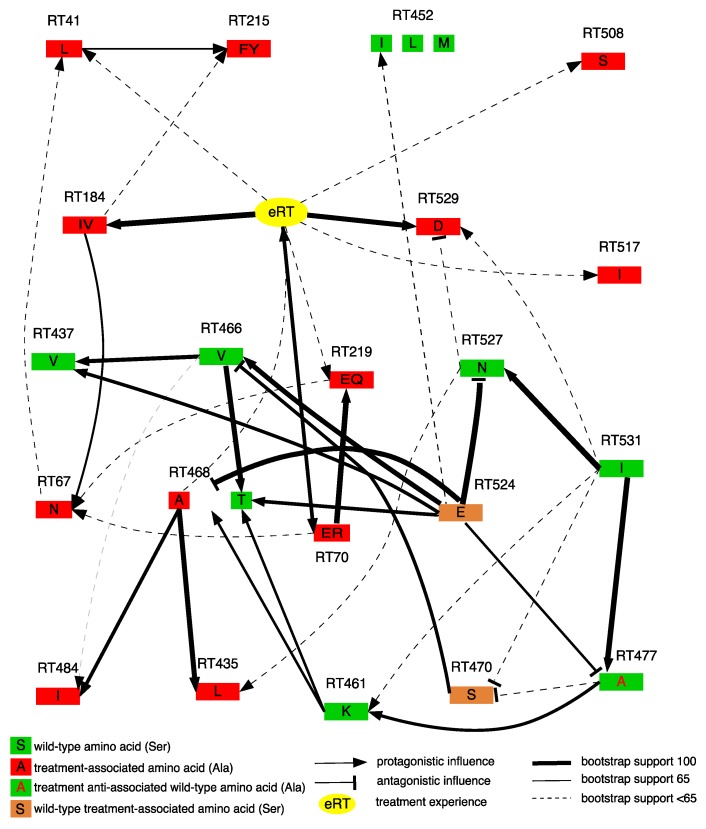
Annotated Bayesian network, based on 500 bootstraps, showing graphically the learned associations between nodes denoting amino acids or treatment experience. An arc (arrows) represents a direct dependency between corresponding nodes and arc thickness is proportional to bootstrap support. An antagonistic arc with a wild type was treated the same as a synergistic arc with mutations at this position. Arc direction has no causal meaning, but may indicate a non-additive multivariate effect.

We explored associations between the presence of natural polymorphisms, the emergence of mutations and NRTI treatment experience using Bayesian network learning. Figure 3 shows that mutations in the RNase H domain did not interact with any known NRTI-elicited mutations. A direct robust interaction was observed between treatment experience (eRT) and known NRTI-elicited mutations M184I/V and K70E/R. A strong interaction also existed between treatment experience and mutation E529D in the RNase H domain, independently of other mutations. Interactions between treatment experience and RNase H domain mutations S468A, A508S and L517I were less robust. RNase H mutation S468A was further strongly connected to other RNase H mutations L484I and A435L. Polymorphisms T470S and Q524E, that increased in prevalence following treatment, did not directly interact with any known NRTI-elicited mutations and/or treatment experience, indicating a potential supporting role.

## 4. Discussion

The role and effects of mutations in the RNase H domain in contributing to NRTI failure have not been fully elucidated, particularly in non-B subtypes, with most available data describing subtype B sequences from developed countries. Here, treatment-related mutations were identified by comparing mutation frequencies in subtype C NRTI treatment-experienced sequences to those in subtype C ART naive sequences. Our findings indicate that several previously identified NRTI drug resistance mutations (435, 483, 491, 519, 530, and 554) of subtype B [7,8,9,11,12] represent the wild-type in subtype C, thereby warranting further characterization of their role. In our cohort, the E529D mutation was significantly more prevalent in sequences from NRTI treated patients, suggesting that it is a treatment related mutation in South African subtype C viruses.

As expected, most of the amino acids in the active site and primer grip site were conserved, as mutations in these regions impact on the functioning of RNase H [7,9]. Previous reports demonstrated by site-directed mutagenesis that mutations (N474, Q475 and Y501) in or near the primer binding site reduced RNase H cleavage specificity and modified the enzyme’s ability to remove the polypurine tract primer. Furthermore, mutations in the RNase H sites overlapping the protease cleavage site caused defects in the virus and malfunctioning of the HIV-1 integrase enzyme [7,9]. Taken together, these findings suggest that any changes in positions closer to these functionally important sites may lead to RNase H and RT enzyme inefficiency.

None of the resistance-associated mutations in RNase H (L469F, K527N, Q509L and K558E/R) identified for subtype B [7] were found to be significant among treated compared to naive participants in this cohort. Codons L469 and K527 were highly polymorphic in both ART-naive and treatment-experienced sequences, with the exception of position K558 that showed a low degree of variability in both treated and treatment naive sequences. Additionally, this study did not observe any association between K558 and thymidine analog mutations (TAMs) and this in contrast with previous studies that showed combination of K558 and TAMs conferring resistance to thymidine analog drugs. Furthermore, mutations at RNase H positions 435, 483, 491, 519, 530, and 554 previously reported to be associated with NRTI drug resistance in subtype B studies were found in both naive and treated sequences at high prevalence, indicating that these are naturally occurring polymorphisms in subtype C. Previous studies have reported that position 469, 470 and 554 are near the RNase H primer grip and active site, suggesting that any changes in these positions could reduce the RNase H activity by indirectly modifying the interaction between the C-terminal of RT and its nucleic acid substrate [12,13]. In combination with TAMs and A371V, Q509L mutation reduced AZT susceptibility about 10- to 50-fold in vitro [19,20]. Overall, our study demonstrated similar subtype difference between subtype B and C in protease and polymerase domains [36], suggesting that these differences could affect replication capacity and cytopathogenicity of subtype C sequences compared to subtype B sequences, or result from a confounding effect of shared history.

Resistance pathways to NRTI treatment were further investigated using Bayesian Network Learning. The presence of RNase H mutation E529D was directly dependent on whether or not the patient received an NRTI, supporting its role in the development of NRTI resistance. Recent work by Barral et al. (2016) also observed a prevalence increase of E529D following treatment administration in subtype C patients in Brazil [8], further supporting our findings. Other RNase H mutations were also connected to treatment experience, although the support for these connections was low. Phylogenetic analyses indicated that the emergence of E529D patients did not result from a shared ancestry but rather from multiple independent selection events. Unfortunately, this study was unable to directly link the presence of E529D with a specific antiretroviral drug since we could not differentiate d4T from AZT users within the treated group. However, the exact role of E529D needs to be further investigated using site-directed mutagenesis as this mutation can either increase phenotypic resistance or restore enzymatic activity.

## 5. Conclusions

This study identified differences in the mutational response of RT to NRTI treatment between subtype B and South African subtype C, with novel mutation E529D, in particular, strongly associated with treatment experience. Results obtained from this study strongly support the need for additional research on the role of the RNase H region in the development of HIV-1 drug resistance, using larger association studies and drug susceptibility assays. Findings from subtype B studies should be re-evaluated to exclude a confounding effect of epidemiology on the identification of mutations.

## Supplementary Materials

The following are available online at www.mdpi.com/1999-4915/9/11/330/s1, Figure S1: Amino Acids distribution in the RNase H Domain Sequences From Treatment Experienced HIV-1 Subtype C Infected Patients. Figure S2: Amino Acids distribution in the RNase H Domain Sequences From Treatment Naïve HIV-1 Subtype. Figure S3: Amino Acids distribution in the RNase H Domain Sequences From Treatment Naïve HIV-1 Subtype B Infected Patients.
