# Supplementary material for: Characterization of Nucleoside Reverse Transcriptase Inhibitor-Associated Mutations in the RNase H Region of HIV-1 Subtype C Infected Individuals
Source: Viruses. 2017 Nov 8;9(11):330. doi: 10.3390/v9110330 (PMC5707537; doi:10.3390/v9110330)
Supplement: Supplementary file 1 [file viruses-09-00330-s001.pdf]

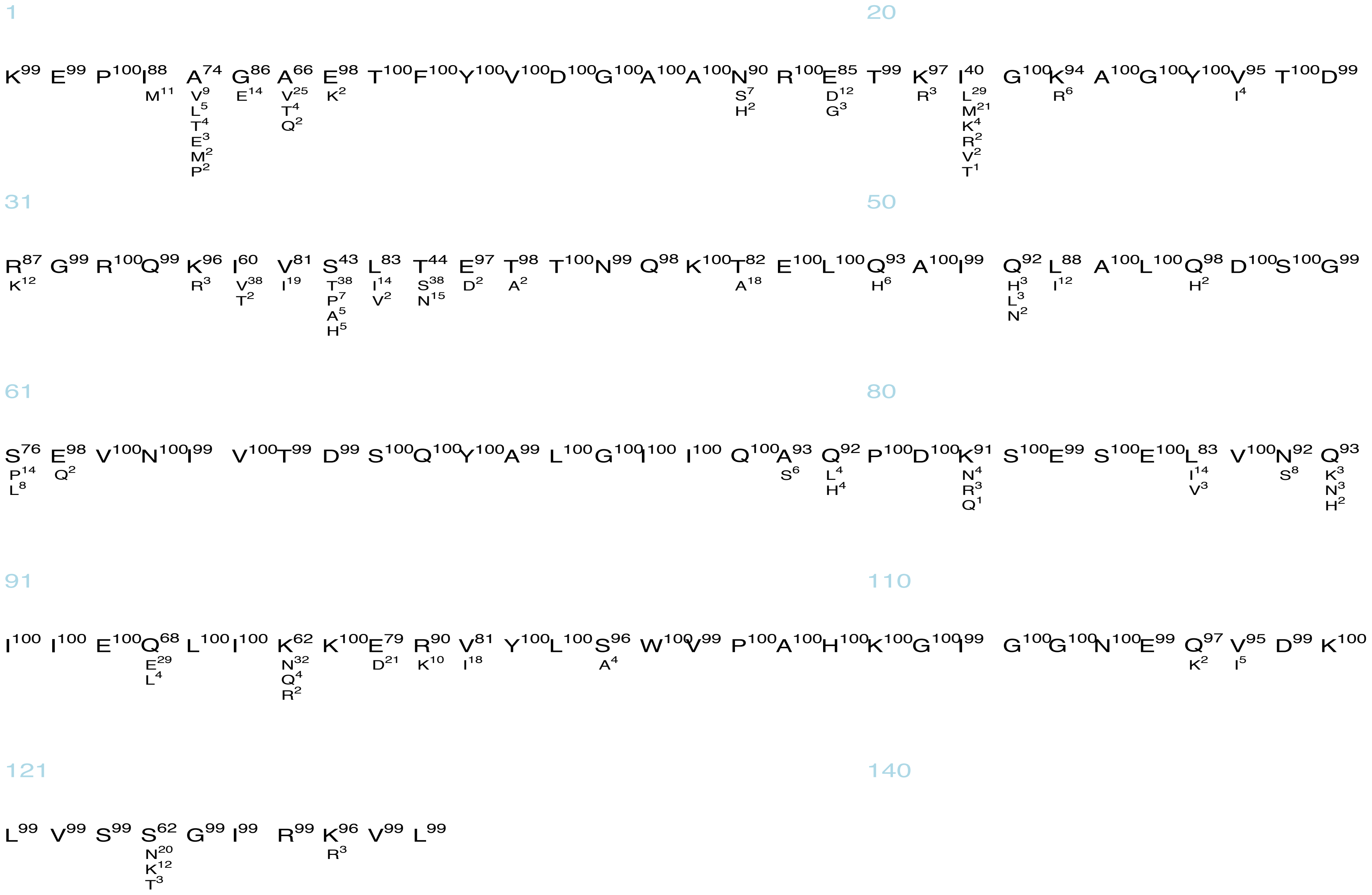

Figure S1. Amino Acids distribution in the RNase H Domain Sequences From Treatment Experienced HIV-1 Subtype C Infected Patients

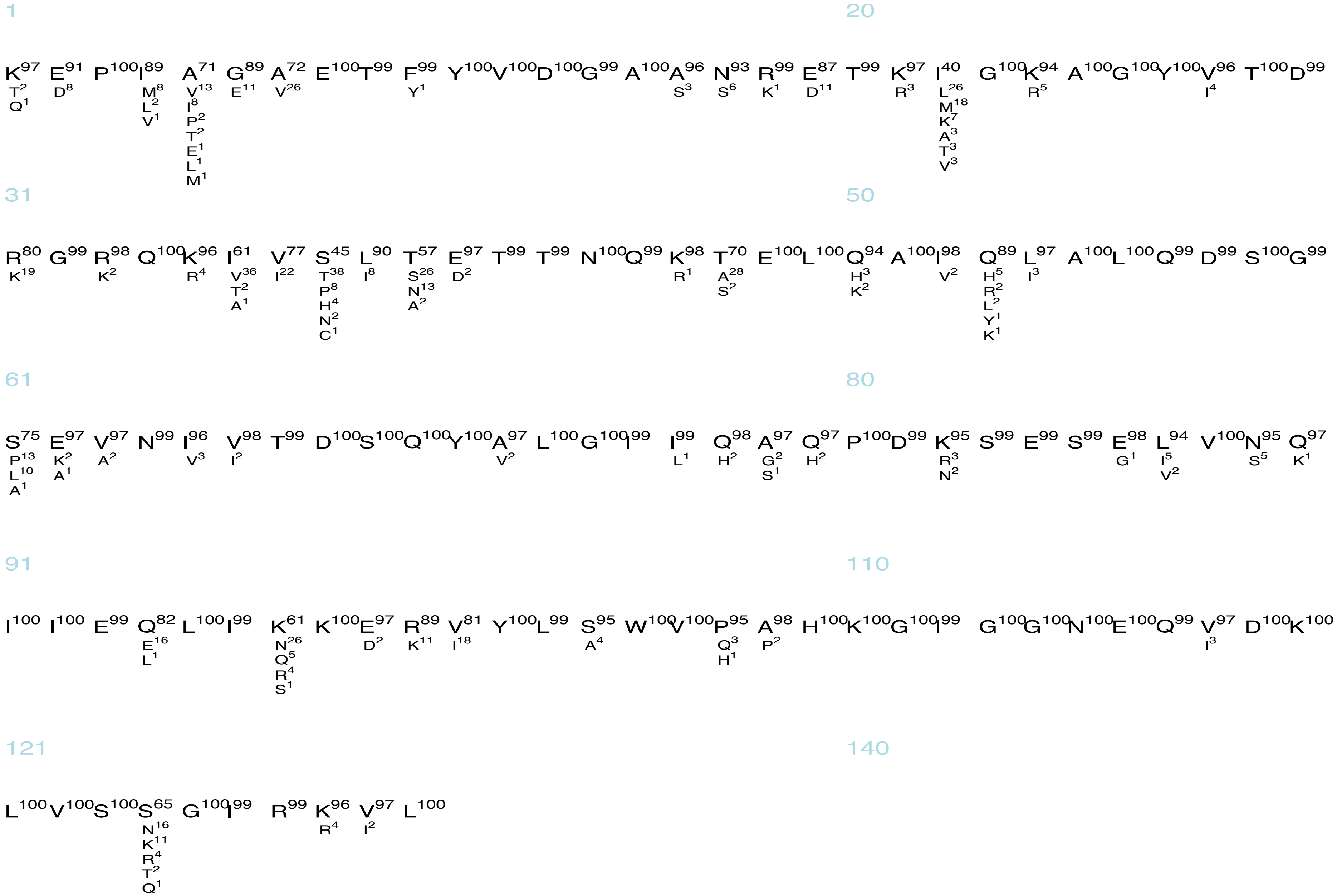

Figure S2. Amino Acids distribution in the RNase H Domain Sequences From Treatment Naïve HIV-1 Subtype C Infected Patients

431																			450										
K^{95}	E^{95}	P^{100}	I^{99}	V^{56}	G^{98}	A^{98}	E^{100}	T^{100}	F^{99}	Y^{100}	V^{100}	D^{100}	G^{100}	A^{100}	A^{97}	N^{95}	R^{99}	E^{92}	T^{98}	K^{95}	L^{85}	G^{100}	K^{94}	A^{100}	G^{100}	Y^{100}	V^{98}	T^{100}	D^{58}
T^{3}	D^{5}			I^{16}	E^{1}	V^{2}									S^{3}	S^{5}		D^{7}	S^{1}	R^{5}	I^{7}		R^{5}				I^{2}		N^{40}
R^{1}				E^{15}																	S^{4}								S^{2}
				A^{8}																	V^{2}								
				L^{2}																									

461																			480										
R^{70}	G^{100}	R^{91}	Q^{100}	K^{99}	V^{95}	V^{82}	S^{59}	L^{92}	T^{87}	D^{98}	T^{100}	T^{99}	N^{100}	Q^{100}	K^{98}	T^{98}	E^{100}	L^{100}	Q^{94}	A^{100}	I^{99}	H^{63}	L^{100}	A^{100}	L^{100}	Q^{99}	D^{100}	S^{100}	G^{98}
K^{29}		K^{9}			I^{3}	I^{18}	P^{22}	I^{7}	A^{6}	N^{1}					Q^{2}	A^{1}			H^{3}			Y^{26}							E^{2}
					A^{2}		T^{17}		N^{3}										E^{2}			N^{5}							
									P^{2}													Q^{4}							
									S^{1}													L^{1}							

491																			510										
L^{68}	E^{99}	V^{100}	N^{100}	I^{99}	V^{100}	T^{100}	D^{100}	S^{100}	Q^{100}	Y^{100}	A^{99}	L^{98}	G^{100}	I^{100}	I^{97}	Q^{98}	A^{99}	Q^{96}	P^{100}	D^{100}	K^{90}	S^{100}	E^{99}	S^{100}	E^{98}	L^{87}	V^{100}	S^{71}	Q^{98}
S^{17}												I^{2}			L^{2}	H^{2}		H^{2}			R^{5}					I^{10}		N^{28}	K^{1}
V^{7}																		K^{1}			Q^{4}					V^{3}			
A^{5}																													
P^{2}																													

521																			540										
I^{100}	I^{100}	E^{100}	Q^{90}	L^{100}	I^{99}	K^{86}	K^{100}	E^{99}	K^{95}	V^{92}	Y^{100}	L^{99}	A^{96}	W^{100}	V^{100}	P^{100}	A^{100}	H^{100}	K^{100}	G^{100}	I^{100}	G^{100}	G^{100}	N^{100}	E^{99}	Q^{97}	V^{86}	D^{100}	K^{98}
			E^{9}			N^{8}		D^{1}	R^{5}	I^{8}		M^{1}	T^{3}													K^{2}	I^{14}		R^{2}
						Q^{3}																							
						R^{2}																							

551																			570
L^{100}	V^{100}	S^{100}	A^{54}	G^{100}	I^{95}	R^{100}	K^{84}	V^{95}	L^{100}										
			S^{20}		V^{5}		R^{16}	I^{5}											
			T^{18}																
			N^{8}																

Figure S3. Amino Acids distribution in the RNase H Domain Sequences From Treatment Naïve HIV-1 Subtype B Infected Patients
